# Multicompartment modeling of protein shedding kinetics during vascularized tumor growth

**DOI:** 10.1038/s41598-020-73866-8

**Published:** 2020-10-07

**Authors:** Gautam B. Machiraju, Parag Mallick, Hermann B. Frieboes

**Affiliations:** 1 Biomedical Informatics Training Program, Department of Biomedical Data Science, Stanford University, Stanford, CA USA; 2 Canary Center at Stanford for Cancer Early Detection, Stanford University, Stanford, CA USA; 3 Department of Radiology, Stanford University School of Medicine, Stanford, CA USA; 4 Department of Bioengineering, University of Louisville, Louisville, KY USA; 5 James Graham Brown Cancer Center, University of Louisville, Louisville, KY USA; 6 Center for Predictive Medicine, University of Louisville, Louisville, KY USA

**Keywords:** Tumour biomarkers, Mathematics and computing

## Abstract

Identification of protein biomarkers for cancer diagnosis and prognosis remains a critical unmet clinical need. A major reason is that the dynamic relationship between proliferating and necrotic cell populations during vascularized tumor growth, and the associated extra- and intra-cellular protein outflux from these populations into blood circulation remains poorly understood. Complementary to experimental efforts, mathematical approaches have been employed to effectively simulate the kinetics of detectable surface proteins (e.g., CA-125) shed into the bloodstream. However, existing models can be difficult to tune and may be unable to capture the dynamics of non-extracellular proteins, such as those shed from necrotic and apoptosing cells. The models may also fail to account for intra-tumoral spatial and microenvironmental heterogeneity. We present a new multi-compartment model to simulate heterogeneously vascularized growing tumors and the corresponding protein outflux. Model parameters can be tuned from histology data, including relative vascular volume, mean vessel diameter, and distance from vasculature to necrotic tissue. The model enables evaluating the difference in shedding rates between extra- and non-extracellular proteins from viable and necrosing cells as a function of heterogeneous vascularization. Simulation results indicate that under certain conditions it is possible for non-extracellular proteins to have superior outflux relative to extracellular proteins. This work contributes towards the goal of cancer biomarker identification by enabling simulation of protein shedding kinetics based on tumor tissue-specific characteristics. Ultimately, we anticipate that models like the one introduced herein will enable examining origins and circulating dynamics of candidate biomarkers, thus facilitating marker selection for validation studies.

## Introduction

Although the search for blood-borne cancer protein biomarkers to improve diagnosis and prognosis has accelerated in recent years, only a handful of candidates have reached clinical application^1–7^. A major reason is that the relationship between protein abundance in tumor tissue and in blood circulation remains poorly characterized. It has proven difficult to link the cell-scale events (e.g., protein shedding into circulation) to the dynamically evolving tissue-scale conditions (e.g., tissue access to vasculature that is changing as a result of proliferating and necrosing tissue). As the cell activity (proliferation and death/necrosis) and the associated protein shedding occur on similar time scales, the detection of proteins in circulation presents primarily a spatial problem, as proteins must persist and diffuse through space from cells to blood under conditions that are continuously changing this space and the access to the blood.

To complement experimental efforts, mathematical modeling and computational simulation techniques have been applied to predict circulating biomarker levels from tissue-scale data^8–13^. Previous continuum-scale modeling of tumor growth has considered the effects of molecular and cellular heterogeneity on tumor spatial progression (e.g.,^14,15^), but not in the context of blood-borne biomarkers. In particular, Gambhir and coworkers developed a 1-compartment model to simulate shedding of the secreted ovarian cancer marker CA-125 into blood plasma^8^. Applying this compartmental approach and its assumption of tumor and healthy cell populations contributing uniformly to shedding, tumor tissue and its vasculature were modeled as singular homogeneous entities without cellular level detail. The strength of this model lies in a small set of parameters that can be easily interpreted and tuned. However, the model over-simplifies the tumor growth process, neglecting intra-tumor heterogeneity due to inhomogeneous vascularization that directly affects marker shedding into the tumor microenvironment and the blood circulation. Additionally, the model lacks the ability to simulate non-extracellular (non-EC) proteins (surface or intracellular in domain). As less than $20\%$ of cellular proteins are secreted^16^, it may be challenging to explore the majority of candidate biomarkers using this approach.

Building upon the work of Gambhir and coworkers^8^, we present a new model that links the levels of protein shedding into circulation to the heterogeneous tumor spatial vascularization and the corresponding proliferating and necrosing cell populations. This is achieved by discretizing tumor tissue into dynamically evolving compartments based on cellular distance from vasculature^17^, effectively bridging from the cellular to the tissue scale, and thus enabling prediction of system-level effects from cellular-scale events. In this model, proteins shed by cells diffuse through interstitial space to reach the vasculature in order to enter the blood circulation. It is well known that incipient tumors begin to slow growth and require sustained angiogenesis to meet the demands of their growing cell populations^18^. Vascularization induces gradients of oxgygen and nutrients in surrounding tissue, imposing cellular stresses such as hypoxia-induced necrosis, which influence shedding of both extracellular and non-extracellular proteins^9^. Intratumoral heterogeneity is induced by cells with higher net growth rates in well oxygenated regions proximal to vasculature, and with lower net growth rates in hypoxic and necrotic regions distal to the blood supply^19^. By simulating the dynamics of tumor development and the spatial variation in cell viability across heterogeneously vascularized tumor tissue, the shedding kinetics of a wide variety of proteins can be described as a function of tumor tissue characteristics. In particular, by enabling an explicit quantification of necrosis, it becomes tractable to model the shedding of the tens of thousands of non-extracellular proteins.

## Results

### A compartmental model to represent protein shedding by heterogeneously oxygenated tumor tissue

**Figure 1:**
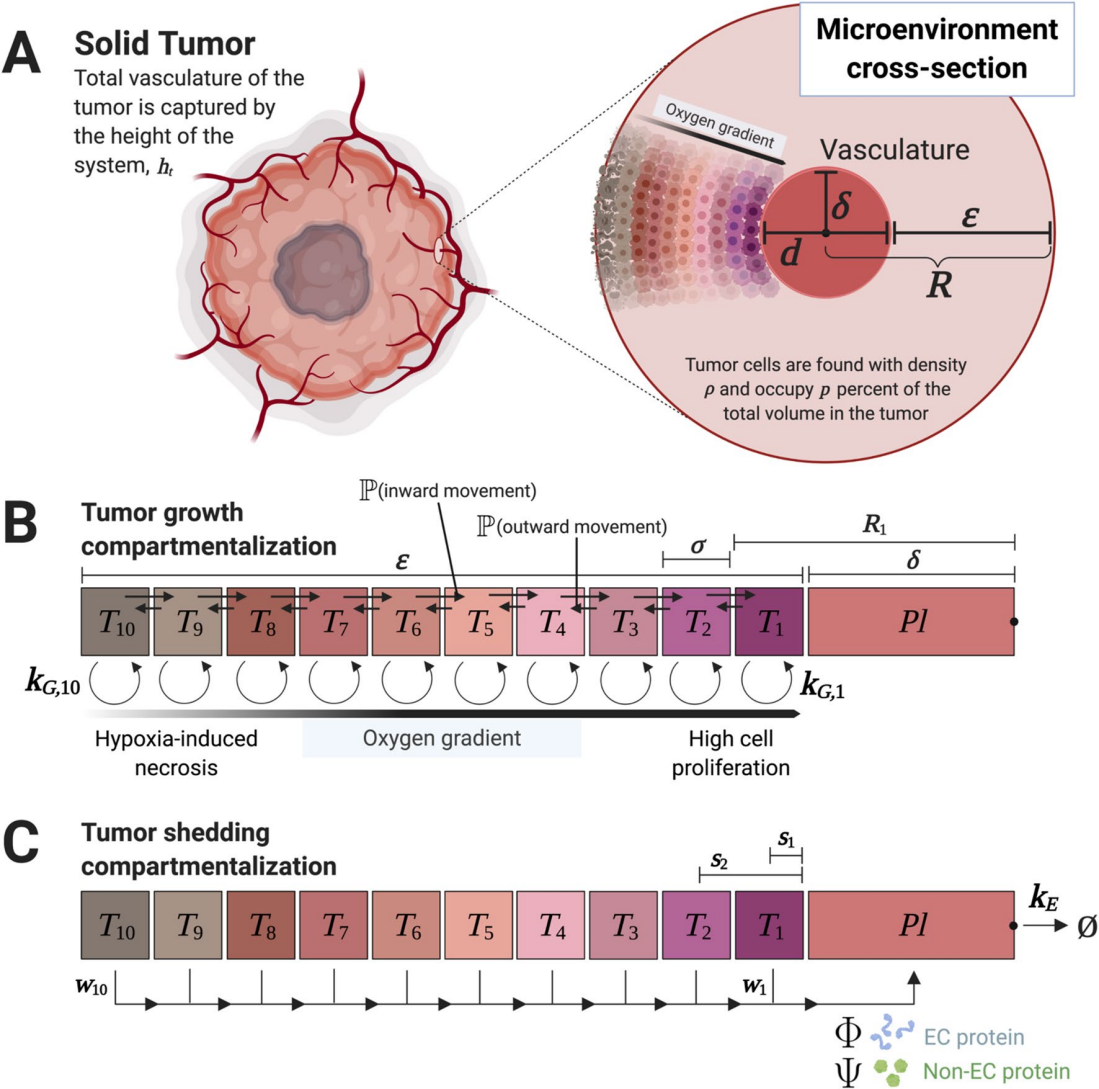
Biological parameters for vascularized tumor growth and compartment model formulation. **(A)** Motivation behind modeling heterogeneous subpopulations in the tumor microenvironment. The model approximates tumor growth as vascularization occurs early in development (abstracting various methods of tumor vascularization, including angiogenesis, vasculogenic mimicry, and microvessel formation). The microenvironment cross-section represents any given 2D neighborhood perpendicular to the vasculature. The vessel diameter *d*, necrotic cuff $\epsilon$, and cylindrical model radius *R* are parameter values derived from cellular spatial constraints obtained from histology images^23^. The radius *R* of the cylindrical model is equal to the combined length of half the mean vessel diameter ($\delta = d/2$) and the necrotic cuff ($\epsilon$). Thus, the cells in the tumor are approximated by the sum of all such 2D cross-sections taken over the length of the total vasculature as governed by $h_t$. Tumor cell subpopulations are defined by their radial distance to proximal vasculature due to heterogeneous access to oxygen. This radial parameterization is discretized with compartments that correspond to each cell subpopulation. Tumor compartments vary in volumes and carrying capacities and are calculated with the recurrence relation of subvolumes from cylindrical shell and spatial constraints described herein. The kinetics of vascularization updates the constraints on compartmental volumes and carrying capacities at each time step. **(B)** Compartment diagram and parameters for tumor growth. Parameter $\sigma$ is the uniformly partitioned thickness of each compartment, set at approximate single-cell diameter of 10 $\mu$m. Compartments $T_i$ all generate cells with their specified birth, death, and net growth rates ($k_{G,i}$) based on their access to oxygen (i.e., distance to vasculature). These compartments thus experience proliferation and necrosis at differing rates, which subsequently leads to differing shedding behaviors. Cell motility with a preference toward oxygenated regions allows for added dynamism of the model (denoted as probabilistic terms between compartments). **(C)** Compartment diagram for protein shedding from tumor cells to plasma. Extracellular protein shedding is dependent on the net proliferative population over the compartments and $\Phi$ (per-cell active shedding rate), while non-extracellular (intracellular and surface) protein shedding is dependent on the instantaneous cell death over the compartments and $\Psi$ (per-cell instantaneous contribution due to turnover). Shedding to plasma, denoted as the *Pl* compartment, is further weighted by distance to vasculature with weights $w_i$ to account for heterogenous diffusion based on distance.

This study focuses on two fundamental coupled features of tumor tissue that impact protein shedding kinetics: cells that proliferate or die (necrose), and heterogeneous vascularization. We design a model to simulate proliferating and necrosing populations. Modeling these two populations distinctly allows for differential shedding rates of extracellular proteins and non-extracellular proteins. Specifically, viable cells dominantly release extracellular proteins, whereas necrosing cells dominantly release intracellular proteins through cell membrane decay and decreased production and secretion of extracellular proteins. Once exported from the cell, these proteins must diffuse through interstitial space to reach the vasculature. Heterogeneous vascularization leads to cell subpopulations experiencing varying levels of oxygen and nutrient availability (Fig. 1A). Differential oxygen and nutrient availability in turn impact cellular proliferation and necrosis, and the associated release of proteins into the interstitium. This allows modeling the difference in shedding rates between extra- and non-extracellular proteins from viable and necrosing cells as a function of heterogeneous vascularization.

To achieve this dynamic representation of protein shedding kinetics, the tumor model is constructed as a series of compartments. At any one time, we assume that each compartment acts as a source of proteins and as a sink for oxygen and nutrients. The whole vasculature within the tumor is represented as a source of oxygen and nutrients and as a sink for proteins. We assume that tumor cells can be grouped into distinct compartments in terms of their distance to vasculature, with each compartment having similar proliferating and necrosing characteristics. Thus, the model virtually *rearranges* tumor tissue into a set of compartments as a function of access to the vasculature (Fig. 1B). The vasculature is represented as a cylindrical (tube) compartment, surrounded by concentric cylindrical (shell) compartments of tumor tissue. Each compartment represents the subpopulation of tumor cells being at any one time at the same distance from vasculature. Inner compartments represent tissue proximal to vasculature, while outer compartments simulate tissue distal to vasculature. Tumor and vascular growth are captured by longitudinally extending these compartments along the cylindrical axis as a function of time.

**Figure 2:**
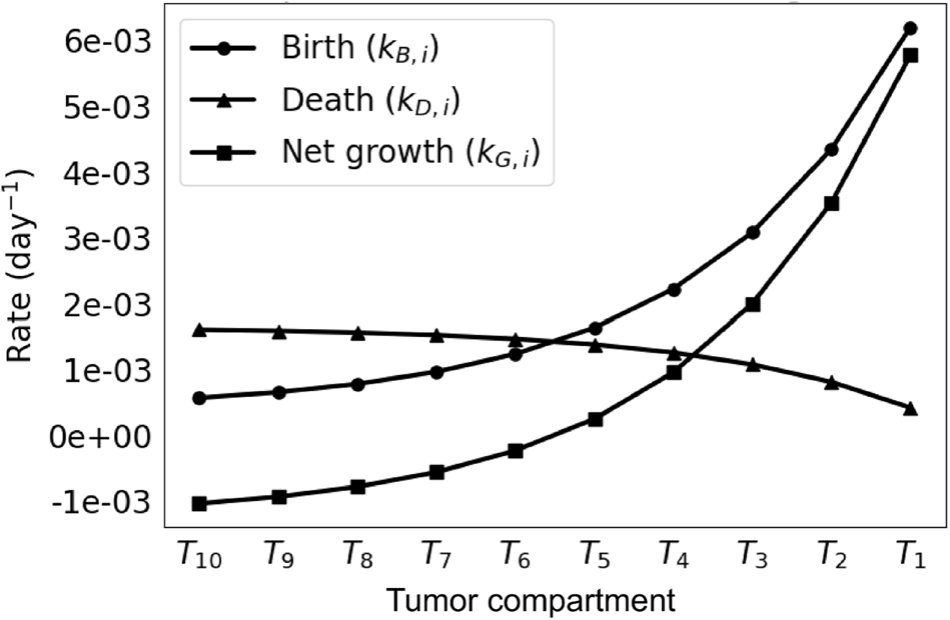
Tumor compartment birth and death rates. Extrapolated rates are derived from cells exposed to varying levels of hypoxic stress^20^. The net growth rates ($k_{G,i}$) are equal to the birth rates ($k_{B,i}$) minus the death rates ($k_{D,i}$). A nonlinear relationship can be observed as a function of compartment number (related to distance from proximal vasculature).

In this manner, vascular-induced intratumoral heterogeneity is represented in the model as a function of radial distance to proximal vasculature, which determines cellular proliferating and death rates in differing oxygenation conditions. It is well known that tumor cells are more proliferative in highly oxygenated conditions and more necrotic in hypoxic conditions, e.g., as shown by cell line data collected from non-small-cell lung cancer cells *in vitro*, which we use here to set compartmental cell birth ($k_B$) and death rates ($k_D$)^20^ (Fig. 2). In turn, the cellular proliferation and necrosis in each compartment affects the respective shedding of extra-cellular and non-extracellular proteins. The proteins are shed into the centrally located vascular compartment according to a weight (*w*) representing diffusivity based on the compartment’s distance to the vasculature (Fig. 1C). With this compartmentalized representation of tumor vascular-induced heterogeneity and its consequences on protein shedding, the model can be formulated with constraints based on observable tumor characteristics. In particular, tissue spatial constraints, e.g., as can be observed from histology^21,22^, are used here as model parameters.

The key parameters that link the spatial relationships in the simulated tumor are chosen as the following (Fig. 1):

*Mean vessel diameter:* The *mean vessel diameter* (MVD; denoted as *d*) is the average diameter of all vascular structures (e.g., blood vessels, capillaries, microvessels, etc.) observed across tumors. The average diameter of larger vessels that sprout microvessels and capillaries has been measured to be approximately 60 $\mu$m across a set of histology slides of retinoblastoma^23^, which is used here as a representative value. The MVD varies between tumors of different tissues.

*Relative vascular volume:* The *relative vascular volume* (RVV) is the ratio of vasculature volume to tumoral volume. This metric has been observed to remain steady during tumor development, e.g., a value of approximately $17\%$^24^ has been measured in mammary adenocarcinoma^25,26^. This value can vary based on the tumor tissue type.

*Necrotic cuff:* The *necrotic cuff* ($\epsilon$) defines a region of viable cells and the space in which a gradient of cell proliferation exists. *In situ*, cells generally fail to grow beyond the diffusion distance of oxygen, approximately 100 $\mu$m^21,23,27^ from proximal vasculature. This metric generally holds for all tumor tissues.

The model implements a longitudinally growing cylindrical tumor tissue of height *h* around a growing vessel. Here, $\epsilon$ is the radius of the cross-sectional model and the MVD constrains the vascular diameter. As tumor vascularization progresses in response to a net release of pro-angiogenic stimuli by tumor tissue, the tissue carrying capacity and growth correspondingly increase. A near-constant RVV allows for the assumption that the MVD and $\epsilon$ of the microenvironment cross-section remain constant as the carrying capacity of the tumor subpopulations increases by increased vascularization.

In this framework, a growing vascularized tumor is represented by *n* discrete tumor compartments $T_i$ of single-cell thickness that shed proteins into the vascular compartment *Pl* (denoting plasma) based on their tumor populations (proliferating or necrotic) and their distance to the vascular compartment.

*Shedding Parameters:* A key component of this model is its ability to capture the shedding of both extracellular and non-extracellular (cell membrane or intracellular) proteins. Prior models exclusively focused on extracellular proteins. However, experimental studies have clearly demonstrated that intracellular components are able to shed to the circulation. This model enables capturing the shedding kinetics of extra- and intra-cellular proteins from proliferating and necrotic cells in heterogeneously vascularized tumor tissue. Key parameters describing the shedding kinetics include $\Psi$, the protein-specific contribution per cell during necrosis, and $\Phi$, the protein-specific shedding rate per cell per day. These two parameters operate in opposition to best capture the differences in shedding dynamics of extracellular vs non-extracellular parameters. Specifically, extracellular proteins will have low values of $\Psi$ and higher values of $\Phi$ that relate to secretion rate. Non-extracellular proteins will have higher values of $\Psi$ calibrated in an abundance-dependent manner, and low values of $\Phi$. Variable $u_i(t)$ is defined as a distance-based relationship between the number of tumor cells and the corresponding compartment’s population and shedding values to capture the diffusion of molecules from varied parts of the tumor into the blood. Variable *q*(*t*) is the final summarized extracellular and non-extracellular protein mass in plasma at time *t*.

### Model calibration

**Figure 3:**
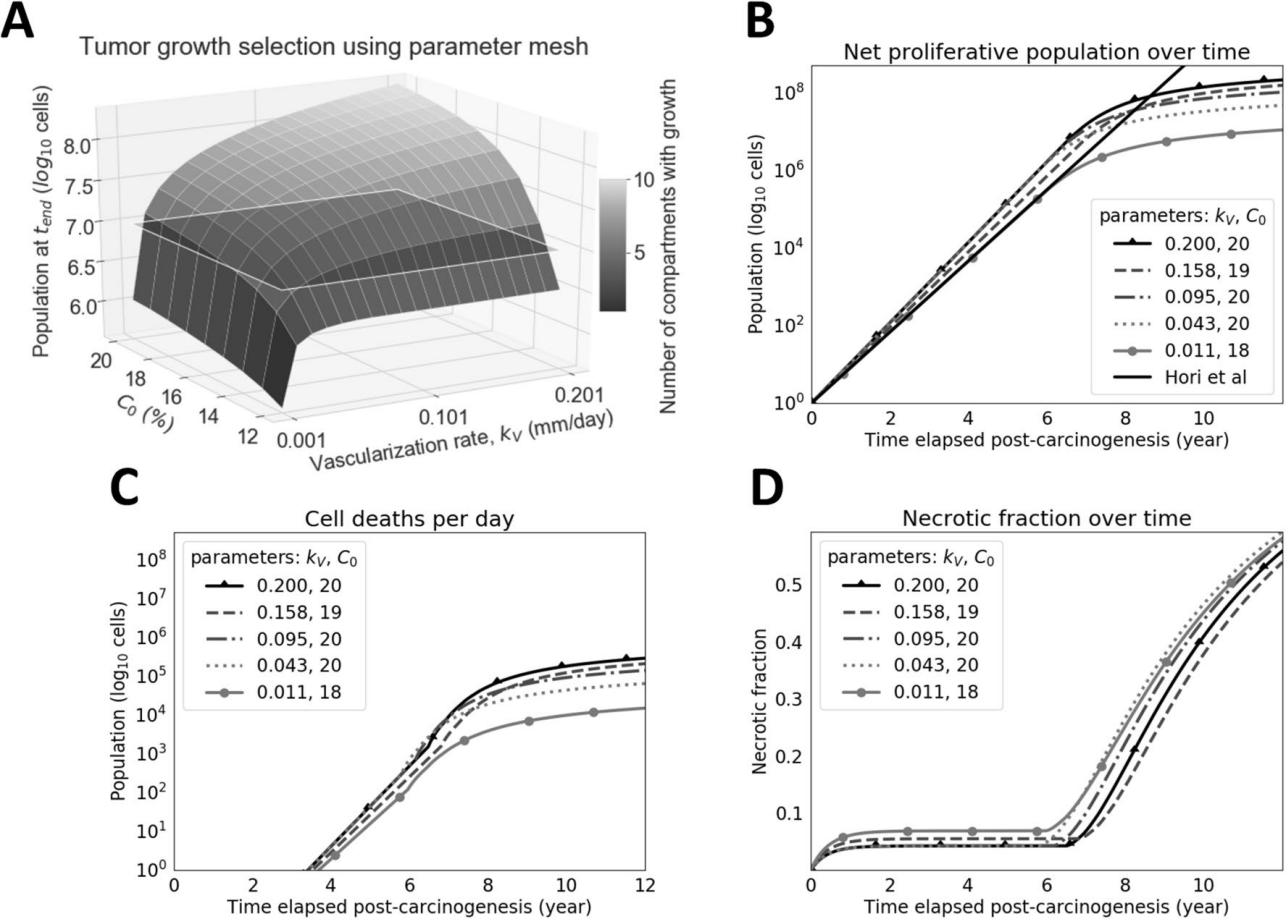
Tumor growth dynamics over time and uniformly-selected simulations over a valid parameter space. **(A)** Selection of biologically-realistic tumors. This surface utilizes both of the parameters $k_V$ (vascularization rate) and $C_0$ (maximum volumetric concentration of oxygen in the tumor, located at vasculature) to form a coordinate mesh along the *x*- and *y*-axes. The *z*-axis represents the final proliferative population at the user-defined maximum iteration for simulation, $t_{end}$. Each tile on the surface represents a simulation with the corresponding parameters on the *x*- and *y*-axes. If the value of $k_V$ is large and $C_0$ is too small, cells primarily stay in compartments near vasculature due to large carrying capacities and little outgrowth. If the value of $k_V$ is too small and $C_0$ is large, carrying capacity is not increased quickly enough and tumor cells die off too quickly to reach distant compartments. Accordingly, valid tumors are defined as those supporting heterogeneous subpopulations and a large overall population. Arbitrary cutoffs were defined to help select for such tumors: those that experience growth in all $n=10$ compartments (indicated by the color bar) and grow to a population of at least $10^7$ cells (represented by translucent plane) at the set maximum iteration of $t_{end}=12$ years. Tumors used for downstream simulation were both above the $10^7$ population plane and with $n=10$ populated compartments, but were also chosen for having the smallest population sizes given the aforementioned selection criteria to select for more necrotic tumors. A sample of five trajectories were taken to generate the lines seen in panels (**B**), (**C**), and (**D**). The trajectory that exceeded the arbitrary cutoffs while maintaining the smallest popuation size was used in all subsequent experiments and figures. **(B)** Valid simulations of tumor proliferation. Simulations are similar to that of Hori et al’s tuned model, but face a slowdown (i.e., greater cell death) in later stages due to unmet demands in vascularization. **(C)** Valid simulations of cell deaths per day. Daily cell death is largely caused by hypoxia-induced necrosis and begins nearly after $t=4$ years when the tumor reaches approximately $10^4$ cells. **(D)** Valid simulations of necrotic fraction represent the ratio of cumulative cell death to the total number of cells accounted for (cumulative cell death and current proliferative population). The necrotic fraction spikes starting at $t=6$ years when an uptick in cell death occurs at the same time, as seen in (**C**). This is an emergent property of the currently used model parameters, as is the case with the trajectories of (**B**) and (**C**).

The first step to employ the model is to choose a set of parameter values that yield tumor growth that is biologically realistic. For this purpose, we chose to match the tumor cell proliferation shown by Hori et al.’s model, which has been calibrated from experimental data with ovarian cancer cells^8^. To run the model over a range of possible parameter values, scans over a parameter set of both the maximum volumetric concentration of oxygen at the vasculature point ($C_0$) and the vascularization rate ($k_V$) were simulated. Parameter $C_0$ helps to determine the range of values for the $n=10$ compartmental net growth rates $k_{G,i}$, helping to shift the range of possible proliferation rates depending on the proliferative capacity of the particular cell type. Because of the large number of parameter combinations, validity cutoffs were arbitrarily defined (e.g., tumor cell proliferation in all $n=10$ compartments, total cell population of greater than $10^7$ cells at $t:=t_{\text {end}}=12$ years) to identify suitable tumor growth simulations. These scanned parameter pairs are presented in Fig. 3. Tumor cell proliferation matching that of Hori et al.’s model was achieved given the model’s linearity and spatial constraints, showing it to be a reasonable spatial extension of their dimensionless model. Using the subset of suitable results, the simulation with maximum growth was selected to evaluate protein shedding profiles.

### Sensitivity of tumor growth and protein shedding to variation in parameter values

**Figure 4:**
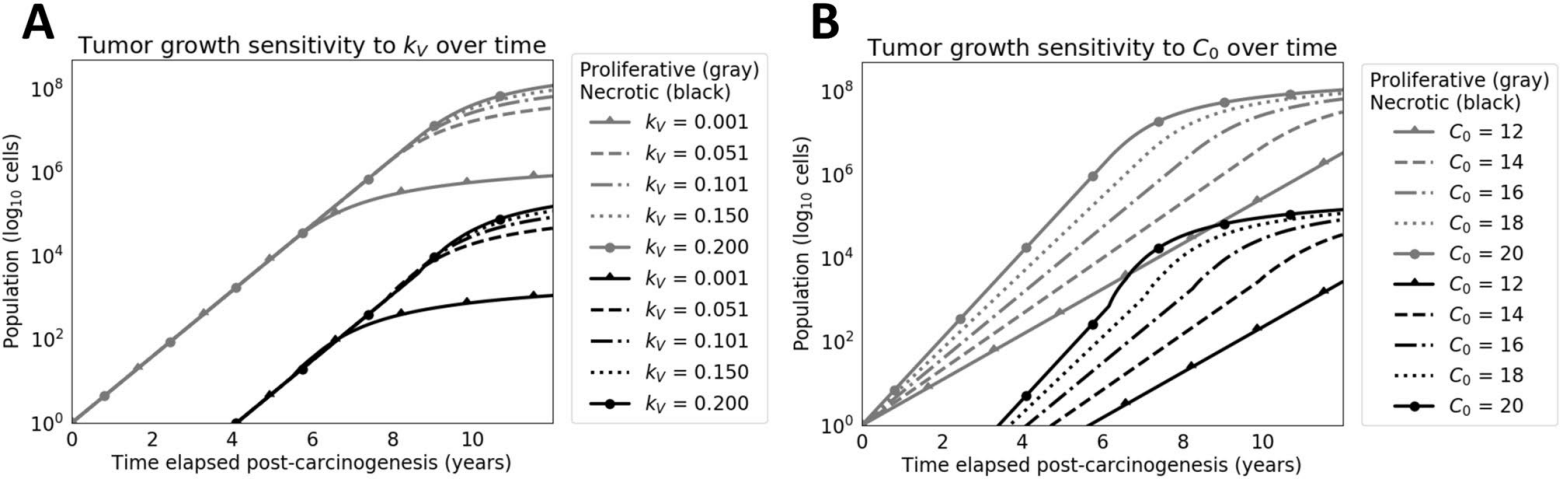
Sensitivity analysis for tumor growth. Parameter values for (**A**) vascularization rate ($k_V$) and (**B**) the maximum volumetric concentration oxygenation at vasculature ($C_0$) interpolated were chosen at five uniformly-spaced values between their respective domain’s hypothesized minimum and maximum values, depending on the parameter of interest. The necrotic population trajectories are colored in black, while the proliferative population trajectories are colored in gray. For each plot, the parameter not undergoing analysis is fixed. Sensitivity of $k_V$ operated on a fixed value of $C_0$=16 and varied values of $k_V$. Sensitivity of $C_0$ operated on a fixed value of $k_V$=0.1005 and varied values of $C_0$. The summary of parameter values used for sensitivity are specified in Table 1, where fixed values are in the *Value(s) Simulated* column. It should be noted that boosting the $C_0$ value shifts the range of $k_{G,i}$ to more positive values, resulting in faster tumor growth. The inflection in the necrotic population (**B**) shows the uptick in cell death after the vascularization rate is outpaced by the growing cell population, causing more cells to experience hypoxic conditions.

**Figure 5:**
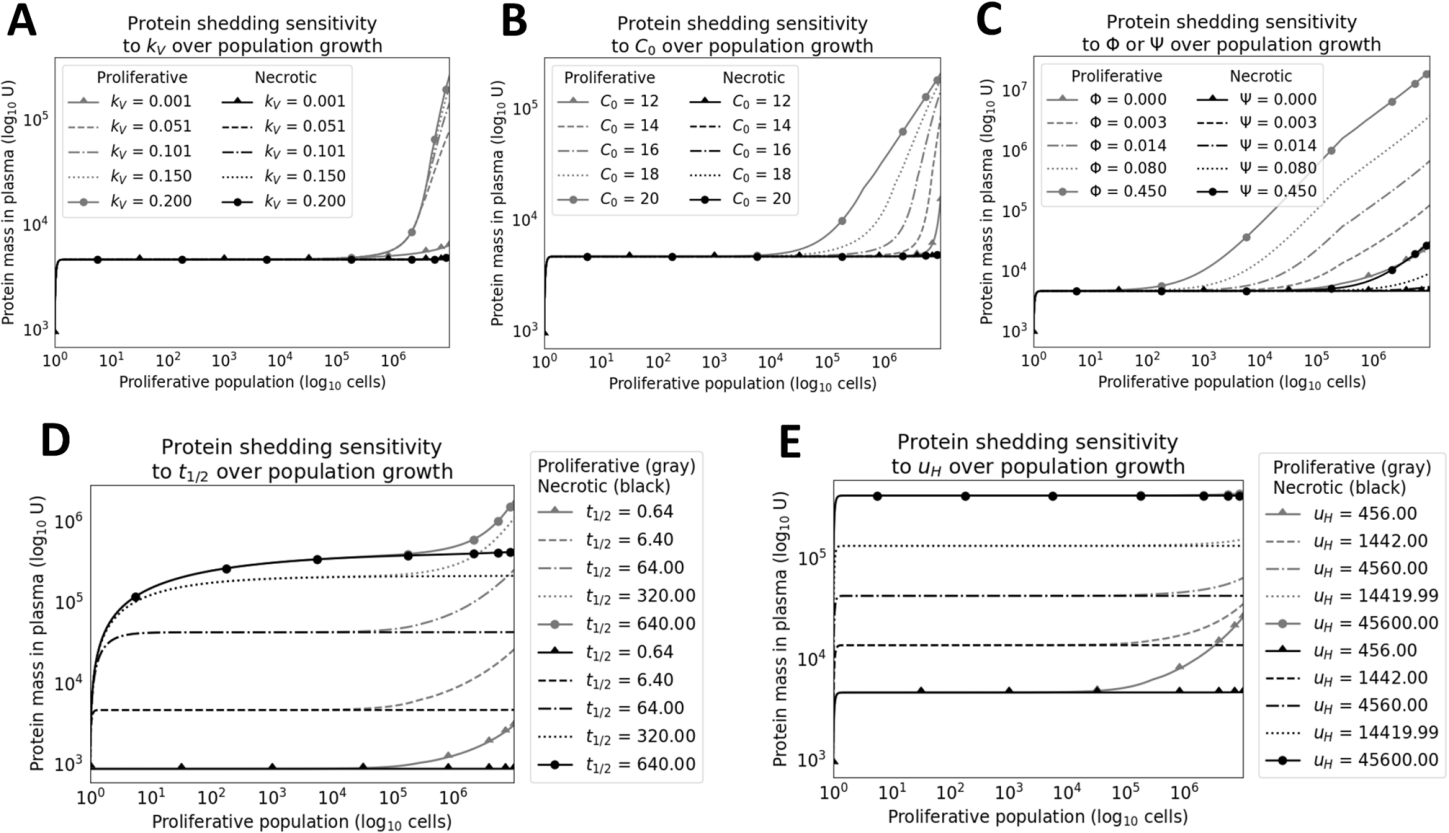
Sensitivity analysis for protein shedding. Parameter values for (**A**) $k_V$, (**B**) $C_0$, (**C**) per-cell influx ($\Phi$ or $\Psi$ depending on the population), (**D**) half-life $t_{1/2}$, and (**E**) the healthy cell shedding influx $u_H$, were chosen at five linear- or log-ordered points between their domain’s hypothesized minimum and maximum values, depending on the parameter of interest. The non-extracellular (non-EC) shedding trajectories are colored in black, while the corresponding extracellular (EC) shedding trajectories are colored in gray. For each plot, the parameter not undergoing analysis is fixed to a hypothetical set ($t_{1/2}$=6.4, $\Phi$=0.00045 or $\Psi$=0.00045 depending on population, and $u_H$=456) acting as baselines for both marker types. The summary of parameter values used for sensitivity are specified in Table 1. This analysis emphasizes the importance of all parameters in shedding of both EC and non-EC makers, as seen by their visibly large log-fold changes in protein mass. Namely, parameter $u_H$ controls the immediate uptick in the trajectory, while parameters $t_{1/2}$ and $\Psi$ or $\Phi$ control the slope of the trajectory after proliferative growth slowdown and the emergence of the necrotic population. Parameter $t_{1/2}$ also controls the initial surge in marker mass before reaching steady state due to controlling the rate of elimination. It should be noted that varying either $k_V$ and $C_0$ alone in the specified parameter ranges does not visibly benefit non-EC shedding since cell death is the source of markers.

Next, the sensitivity of the calibrated model to variation in its parameter values was evaluated. Running these analyses over the full set of parameters introduced by the model, the influence of each parameter on tumor growth (Fig. 4) and protein shedding (Fig. 5) was evaluated for both non-extracellular (non-EC) and extracellular (EC) proteins. These studies examined how stable the model was to variations in or mis-estimations of parameters. For tumor growth, great sensitivity is seen to maximum oxygenation concentration $C_0$ and vascularization rate $k_V$. Parameter $C_0$ modulates the slope of total tumor growth since the parameter set of local net growth rates is shifted by the interpolation function as described in Methods. As a result, constituent compartments are assigned local net growth rates that are shifted accordingly. Parameter $k_V$ appears to enforce a ceiling function for the total tumor population after it outpaces vascularization and necrosis occurs at higher rates. For protein shedding, $t_{1/2}$ (protein half life in blood), $C_0$, and $\Psi$ or $\Phi$ all carry early influence on model performance by large orders of magnitude given realistic ranges specific to each parameter. One may consider both $k_V$ and $C_0$ hyperparameters tuned to a particular tumor, with considerations such as cell and tissue type, while $\Phi$ or $\Psi$ and $t_{1/2}$ are parameters that can be estimated *a priori* for a shedding protein of interest.

For these analyses, the performance of simulated non-extracellular and extracellular proteins was compared. Non-extracellular trajectories tended to be lower and start later than extracellular protein trajectories. This difference stems from the source of non-extracellular proteins being dominated by necrotic cells. Accordingly, the relatively small size of the necrotic population, as compared to that of the proliferative population (Fig. 3), early in tumor development leads to a lag in the shedding of non-extracellular proteins. Despite this lag, our analysis showing overlapping trajectories confirms that non-extracellular proteins can be accessible in the circulation and may reach abundances that exceed those of some extracellular proteins.

### Protein shedding as a function of tumor cell proliferation and necrosis

**Figure 6:**
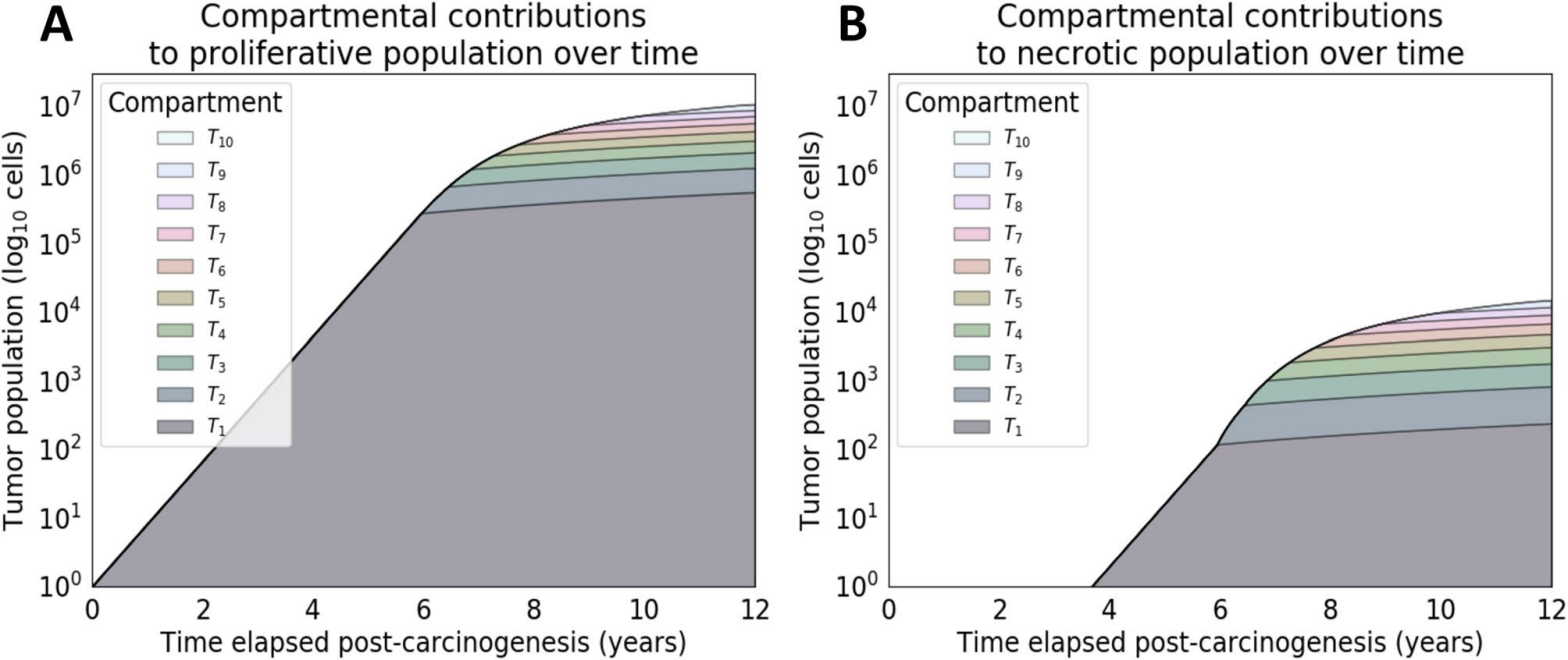
Compartmental contributions toward tumor growth. Stack plot of the per-compartment (**A**) net proliferative and (**B**) necrotic populations over time. The tumor growth run used was programmatically selected for having both moderate tumor growth and necrosis, with the process of model selection seen in Fig. 1. The original exponential trajectory seen from 0–6 years slows down at approximately 6 years into the simulation due to vascularization failing to keep up with the growing metabolic demands of the tumor. This inflection is an artifact of the model’s assumed linear vascularization rate, i.e., the coupled mono-exponential growth models are limited by a linear ceiling. The majority of cells live and die in oxygenated compartments. However, there is a relatively larger contribution to cell death (**B**) from more distant compartments due to their very high death rates.

**Figure 7:**
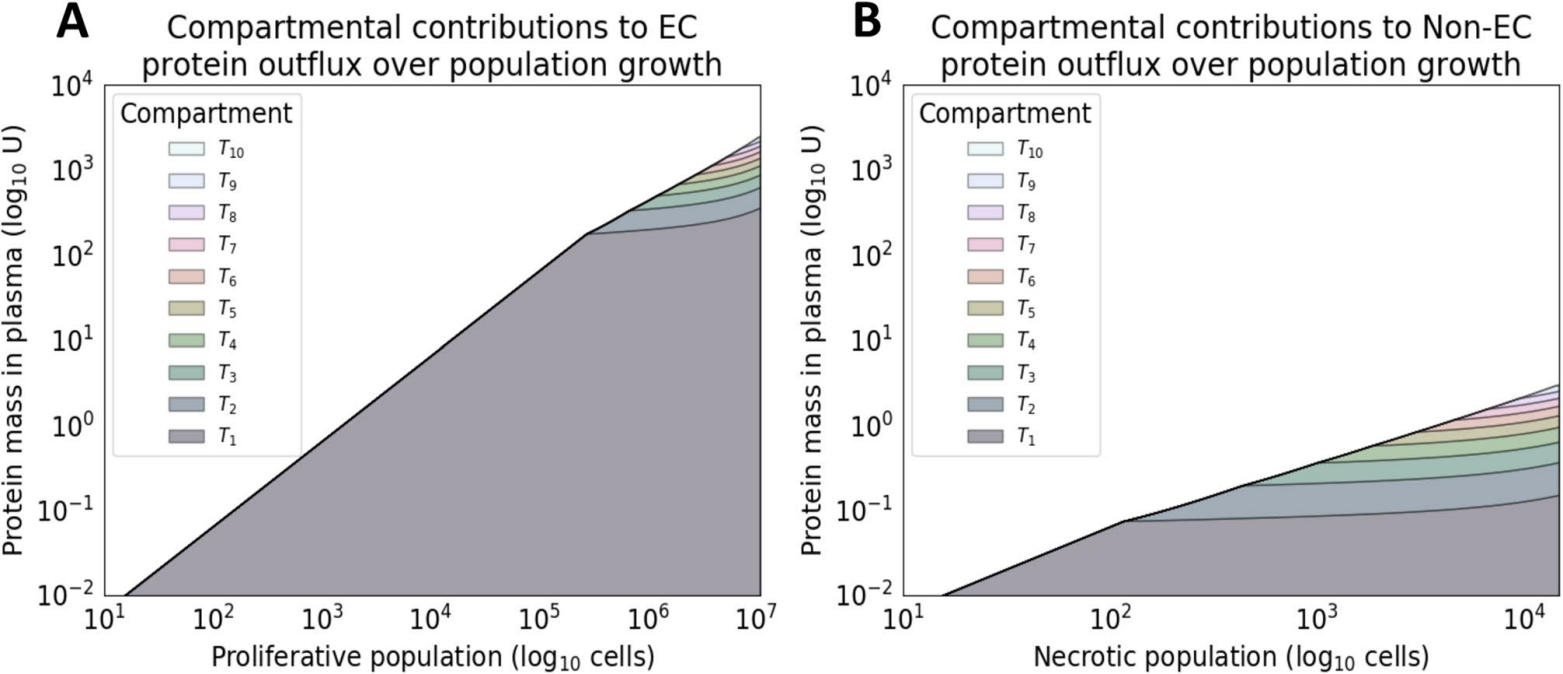
Compartmental contributions toward protein shedding. Stack plot of the per-compartment outflux (or vasculature influx) of (**A**) extracellular (EC) and (**B**) non-extracellular (non-EC) protein mass over time. The slowdown occurs due to the compartmental populations experiencing a limit in carrying capacity (based on vascularization) after the proliferative population reaches approximately $10^5$ cells. Shedding was simulated on the same tumor growth run used for Fig. 6, which was programmatically selected for having both moderate growth and necrosis. The parameter values of the simulated proteins were kept entirely identical, where specifically healthy cell influx ($u_H$), and half-life ($t_{1/2}$) are the same. Furthermore, parameter values of $\Phi =\Psi$ were also assumed in this setting for ease of comparison. Once again, the relatively larger ratio of distant cell contribution to non-EC shedding (**B**) due to higher rates of cell death is visible here. The overall trajectory is near-linear (on log-scale) for both protein localizations.

Next, we evaluated the capability of the model to simulate tumor proliferative and necrotic population evolution in time and the corresponding protein outflux. A goal of the proposed model is to provide a mechanistic understanding of the shedding kinetics of tumor cell subpopulations. Figure 6 shows the per-compartment populations of both proliferative and necrotic cells. In panel (B), an inflection at approximately six years of simulation time is noted. This is due to necrotic cell populations outpacing the rate of vascularization. Spatially, and in the context of the presented model, cells begin to occupy more necrotic compartments (distal to vasculature) due to the linear vascularization function. Figure 7 shows a similar effect for the cell subpopulations’ marker outflux and contributions to overall shedding. Changes in rate for both panel (A) and (B) can be seen at approximately $10^5$ and $10^2$ cells respectively, indicating that the simulated tumor reaches a steady-state in marker shedding at later stages.

### Model validation

**Figure 8:**
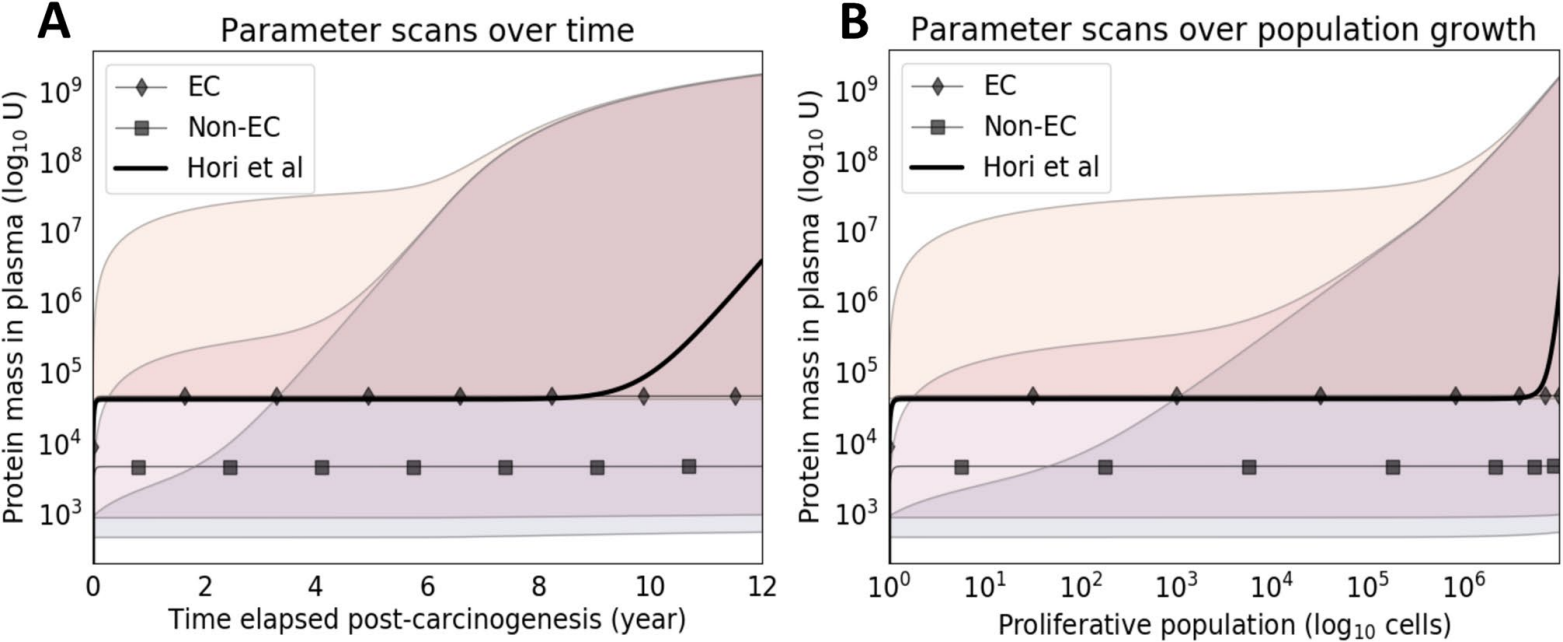
Parameter scanning for non-extracellular proteins. Scans over protein parameters identifies cases when non-extracellular proteins outperform extracellular ones. Hypothetical extracellular (EC) and non-extracellular (non-EC) proteins are simulated and denoted by the lines embellished with diamonds and squares, respectively. Combinatorial scans over the parameters $\Psi$, $u_H$, and $t_{1/2}$ are calculated. As discussed further in Fig. 5, parameter $u_H$ (set to values 4.56e+00, 4.56e+02, 4.56e+04) appears to control the immediate uptick in the trajectory, which can be interpreted as the initial levels of protein mass in circulation. Parameters $t_{1/2}$ (set to values 0.64, 20.24, 640) and $\Psi$ (set to values 1.423e−02, 2.531e+00, 4.500e+02) appear to control the slope of the trajectory, which is especially visible after tumor growth slowdown and emergent necrotic population uptick. The minimum and maximum trajectories are taken for each value of $u_H$ scanned, resulting in shaded regions that define the operating dynamic range of shedding for the scanned parameter space of the other two parameter values. Specifically, the lavender-tinted, orange-tinted, and yellow-tinted regions are the dynamic ranges operating on varying the aforementioned $t_{1/2}$ and $\Psi$ values along with set $u_H$=4.56e+00, $u_H$=4.56e+02, $u_H$=4.56e+04, respectively. Due to a monotonic increase in protein mass with respect to $t_{1/2}$ and $\Psi$, given a set value of $u_H$, the lower and upper bounds of dynamic ranges are always achieved with the smallest and largest values of $t_{1/2}$ (0.64 and 640) and $\Psi$ (1.423e−02 and 0.423e−02), respectively. The crossover of dynamic ranges (tinted regions) indicates parameter operating regions where the initial boost in protein mass from $u_H$ is made up by the other parameters. Results are presented (**A**) with respect to time and (**B**) tumor population. The solid black line represents the Hori et al tuned shedding (using its underlying population growth model), while the line marked EC represents the shedding by the model proposed in this study, with the same parameter values as the Hori model. The end-behavior discrepancy between the two trajectories is most likely due to the differing tumor growth equations and associated assumptions.

As an initial validation, the model-simulated protein shedding was compared to that of Hori et al.’s experimentally tuned model (Fig. 8). The results show that the proposed model can be used as a spatial extension of one-compartment models, indicating similar trajectories between the proposed model and the Hori shedding model for an extracellular marker such as CA-125. However, there is a notable difference in late-simulation behavior due to the linear vascularization function growing too slowly for the total tumor population. This slowdown causes increased necrosis and increased non-extracellular shedding, which is a model-derived hypothesis to be tested *in vivo*. With the selected extracellular protein features from CA-125, hypothetical non-extracellular protein shedding trajectories were generated. The comparison against this extracellular marker was performed via parameter scanning over possible values of protein-specific parameters $\Psi$, $u_{H}$ (healthy cell influx rate of protein mass), and $t_{1/2}$. The colored regions show the dynamic range of trajectories for each value of $u_H$, which effectively sets the basal mass that the growing tumor builds upon. The dynamic range is determined by the other two protein-specific parameters controlling plasma influx ($\Psi$) and elimination (as determined by $t_{1/2}$). Overall, this analysis indicates that non-extracellular proteins may outperform an extracellular protein for blood-based detection given boosted parameter values, highlighting the need for increased study of non-extracellular proteins for cancer biomarker discovery.

## Discussion

This study implements a novel spatial modeling approach that enables *in silico* simulation of extracellular and non-extracellular protein shedding by proliferating and necrosing cell populations in vascularized tumors. The model may be useful for describing the origins of non-extracellular biomarkers in the blood, and creating a scalable framework for asking questions about the impact of environmental heterogeneity on biomarker shedding. These questions are critical, as accessing non-extracellular biomarkers opens up the possibility for thousands of additional proteins to be potential biomarkers. In addition, prior to the development of this model, to the best of our knowledge there has been no straightforward way to link variation in tumor vascularization to marker shedding. A key potential utility of the model will be in comparing the behavior of different candidate markers to each other for the purposes of selecting which markers to carry forward into validation studies. While the identification of such putative markers is beyond the scope of this study, we demonstrate in Fig. 8, that there could be non-extracellular proteins whose circulating abundance can exceed that of extracellular proteins. Individual proteins represent trajectories within these windows that can be directly compared.

Hori & Gambhir^8^ proposed a general model to evaluate biomarkers shed by tumor and host tissue, without distinction as to their intra- or extra-cellular origin, in order to determine the limits of tumor detection from biomarker levels in blood circulation. The model does not consider tumor-specific characteristics, such as vascularization parameters, which can greatly influence the amount and type of biomarker outflux into circulation. Similar to Hori & Gambhir’s model, this study employs a mono-exponential function to simulate tumor growth. Unlike their study, here we are interested in evaluating both non-extracellular and extracellular protein shedding kinetics during this growth, as a function of the tumor tissue characteristics. The model can be tuned by evaluation of histology data, including relative vascular volume, mean vessel diameter, and distance from vasculature to necrotic tissue. This information is used to simulate the dynamic interaction between tumor tissue and vascularization, which determines biomarker outflux into circulation.

The model discretizes cell populations sharing similar oxygenation as a function of distance to vasculature into interconnected compartments. To bypass the spatiotemporal complexities of vascularization, these compartments are in the model assigned increased carrying capacities as the tumor grows. The spatial representation is achieved through the use of concentric cylindrical compartments and, correspondingly, use of the cylindrical parameterization of radial distance to a central vascular compartment. Due to this multi-compartment approach, approximate solutions to difference equations are utilized because (*i*) smaller granularity than at the single day level is not needed, (*ii*) the time-scales of interest are large, and (*iii*) cellular proliferation in the tumor microenvironment is highly dynamic. For a given tissue type and its corresponding growth rates, the model can compute population trajectories along with the corresponding shedding kinetics for a number of proteins of interest. Consequently, this approach enables the comparison of the shedding kinetics of multiple proteins at once given a tumor simulation calibrated to particular tissue characteristics. The implementation is computationally low-cost, providing for spatial modeling without the need to solve coupled partial differential equations^21^ or to represent individual cellular agents^28^. Due to the model’s assumption of coupling between tumor (proliferative and dead) cell populations and plasma protein concentration, as well as an assumed lack of feedback from the plasma and tumor compartments, the tumor growth and protein shedding systems of equations can be computed in series. This computationally efficient methodology leverages *local behavior* of intratumoral dynamics to simulate the protein shedding dynamics of whole tumors. Accordingly, the proposed model offers a novel framework for rapid simulations of tumor shedding with large-scale comparisons between proteins of interest, with the potential to provide insight into patient-specific tumor conditions.

For simplicity, the model employs a standard mono-exponential growth function to describe compartmental growth, yielding relatively small parameter sets both when generating estimators and when calibrating the model. Other growth functions could be explored. Moreover, dying cells (e.g., via necrosis or apoptosis) at each time step do not take up physical space towards the capacity of the compartment and, instead, are instantaneously cleared out of the system. Future tuned parameter sets of net growth rates will need to account for this assumption of space availability. Further, the model parameterization scheme presents certain limitations. The model assumes that cell are sourced from oxygenated compartments at time $t=0$, but in reality there is some *a priori* structure for populations before new vasculature enters the system through angiogenesis or vascular recruitment. Additionally, cell death is not solely caused by necrosis; while increased death rates in less oxygenated compartments imply hypoxia-induced necrosis, the proportion of cell death caused by necrosis as opposed to scheduled cell death is unknown. Furthermore, the vascularization function is currently assumed linear with respect to time. While a density dependent function would reflect angiogenic factors having a role in angiogenesis, a simpler model was chosen. Figure 8 corroborates the need for density-dependent vascularization and faster tumor growth at later stages, as shown by the discrepancy of the presented model and the tuned model by Hori et al. This choice of function controls the rate at which cells can proliferate due to increased carrying capacity. Tuning is required to ensure that the function is not too fast (i.e., large-scale necrosis is delayed) nor too slow (i.e., the tumor fails to achieve a large enough population over time).

Values for the model parameters were primarily chosen from the literature (Table 1). In particular, net tumoral growth, birth, and death rates were tuned to previously-obtained experimental data by us and by others^18^, serving as order of magnitude approximations. The MVD (and resulting volume of vasculature) was chosen as an approximate baseline. Additionally, $k_V$ was itself tuned to achieve a similar growth trajectory as presented in Hori et al. These tumor-specific values should be tuned to a particular tissue type of interest. In order to further tune and validate the proposed model, future work will aim to grow *in vivo* xenografts composed of cell types that shed selected proteins of interest. Known protein tumor kinetics will need to be used to validate model predictions and to verify its utility in order to bring it closer to clinical application. With repeated blood sampling and serial measurements (similar to studies such as Li et al.^29^), the shared tissue-specific model parameters (e.g., growth and vascularization rates) can be tuned to the desired tissue type. After such tuning, protein-specific parameters ($\Phi$, $\Psi$, $u_H$, and $t_{1/2}$) can either be estimated using approaches that average over sampling time or calculated via mass-balance equations once xenografts have nearly reached carrying capacity and steady state is assumed. Lastly, future work will seek to build statistical learning models to predict protein-specific parameters^30,31^ from proteomic features, publicly-available datasets^32^, and steady state values in order to fully enable large-scale viability studies and prioritization of candidate biomarkers.

**Table 1:** Model parameters

Parameter	Description (units)	Value(s) simulated	Range(s) simulated	References
$\mathrm {\Delta }t$	Approximation time step (day)	1	-	-
$k_{G,i}$	Net tumoral growth rate of compartment $T_{i}$ (day$^{\mathrm {-1}})$	See Eq. (17)	Calculated from $k_{B,i}$ and $k_{D,i}$	–
$k_{B,i}$	Tumoral birth rate of compartment $T_{i}$ (day$^{\mathrm {-1}})$	See Eq. (17)	[7.8e−4, 8.2e−3] as original range	^18^
$k_{D,i}$	Tumoral death rate of compartment $T_{i}$ (day$^{\mathrm {-1}})$	See Eq. (17)	[0, 1.6e−3] as original range	^18^
$P_{0}$	Initial number of proliferating tumor cells (cell)	1	–	–
*EC*	Cellular localization of protein	{0, 1}	–	–
$w_{i}$	Distance-based diffusion weight for compartment $T_{i}$	See Eq. (13)	–	–
$\mathrm {\Psi }$	Protein-specific contribution per cell (U/cell)	4.5e-4$^{\mathrm {\# \$ }}$	[1.4e−2, 4.5e2]$^{\mathrm {\$ }}$, [4.5e−4, 4.5e−1]$^{\mathrm {+}}$	–
$\mathrm {\Phi }$	Protein-specific shedding rate per cell (U cell$^{\mathrm {-1}}$ day$^{\mathrm {-1}})$	4.5e−4$^{\mathrm {\# }}$, 4.5e−6$^{\mathrm {\$ }}$	-	^8^
$t_{1/2}$	Blood half-life of protein (day)	6.4	[0.64, 6400]$^{+}$	^8^
$k_{E}$	Elimination rate of protein from plasma (day$^{\mathrm {-1}})$	–	Calculated from $\ln (2)/{t}_{1/2}$	^8^
$$C_{0}$$	Maximum volumetric concentration of oxygen in the tumor (%)	16$$^{\mathrm { \& }}$$	^12,20^$$^{{*+ \& }}$$	^18^
$u_{H,t}$	Healthy cell basal shedding influx; assumed constant (U/day)	4.56e2, 4.56e3$^{{\$ }}$	[4.56e1, 4.56e5]$^{\mathrm {+}}$	^8^
$k_{V}$	Vascularization rate (day$^{\mathrm {-1}})$	0.101$^{\mathrm { \& }}$	[1e-3, 2e-1]$^{{*+ \& }}$	–
*p*	Percent of total tumor volume occupied by tumor cells	0.2	–	^8^
$\rho$	Tumor cell density (cell/mm$^{\mathrm {3}})$	1e6	–	^8^
$\sigma$	Partitioning resolution, or width, of compartments ($\mu$m)	10	–	–
$\epsilon$	Necrotic cuff ($\mu$m)	100	–	^15,19,20^
*d*	Mean vessel diameter (MVD) ($\mu$m)	60	–	^19^
$r_{1/2}$	Radial distance half-life of oxygen	0.018	–	^18^

*U* denotes arbitrary units. It is assumed that $$\Psi$$ is multiple orders of magnitude larger than $$\Phi$$. Original ranges signify those found in the literature before any interpolation. Key: * Values/ranges simulated for valid tumor growth grid search (Fig. 3). & Values/ranges simulated for tumor growth sensitivity analyses (Fig. 4). + Values/ranges simulated for marker shedding sensitivity analyses (Fig. 5). # Values/ranges simulated for parameter scan comparisons between EC and non-EC shedding (Fig. 8). $ Values/ranges simulated for CA-125 shedding simulations (Fig. 8).

## Methods

### Proliferative and necrosing cell populations

To describe proliferation of a tumor cell population *in vivo*, a simple mono-exponential growth model is employed:

1 $$\begin{aligned} \frac{\hbox {d}P(t)}{\hbox {d}t} = k_{G} \cdot P(t) \end{aligned}$$

where, *P*(*t*) is proliferative population at time $t \in {\mathbb {R}}_{\ge 0}$ and $0 \le k_G \le 1$ is net tumoral growth rate (Table 1). For model flexibility and cellular dynamics at each time step, difference equations are used at $\Delta t {:}=1$ day intervals to form the Malthusian expression, $P_{t+1} = P_t + \Delta P$. Through this use of a sufficiently small $\Delta t$, Eq. (1)’s differential equation is converted to Eq. (2) via a forward Euler finite difference approximation:

2 $$\begin{aligned} \frac{\mathrm {d}P(t)}{\mathrm {d}t}&= k_G \cdot P(t) \nonumber \\ \frac{P_{t+1} - P_t}{\Delta t}&\approx k_G \cdot P_t \nonumber \\ \implies P_{t+1}&\approx (1+k_G \Delta t) \cdot P_t \end{aligned}$$

where $P_t$ is the net proliferative population at discrete time point $t \in {\mathbb {N}}$ and $\Delta t = 1$ day is the time step taken for this approximation. The initial condition is set to $P_t := 1$ to signify the number of tumor cells that initiate tumorigenesis. In order to approximate cell death at time *t*, the net tumoral growth rate $k_G$ is decomposed into its constituent birth rate $k_B$ and death rate $k_D$:

3 $$\begin{aligned} k_G \triangleq k_B - k_D \end{aligned}$$

Using the above definition of net growth rate, the terms in Eq. (2) are expanded to define $B_t$ and $D_t$, the amount of cell birth and death at time *t*, respectively:

4 $$\begin{aligned} P_{t+1}&\approx (1 + k_G \Delta t) \cdot P_t = [1 + (k_B - k_D) \Delta t] \cdot P_t \nonumber \\&\approx P_t + \underbrace{k_B \Delta t P_t}_{B_t} - \underbrace{k_D \Delta t P_t }_{D_t} \nonumber \\ \implies D_t&\approx k_D \Delta t \cdot P_t \end{aligned}$$

where $D_t$ is the instantaneous cell death at discrete time *t*, encapsulating both regularly-timed cell death (i.e., apoptosis) and death by injury (e.g., hypoxia-induced necrosis). However for simplicity, $D_t$ is used an approximation of intratumoral necrosis, since this phenomenon governs much of the cell death in hypoxic regions^33^. This formulation for mono-exponential tumor growth and corresponding cell death will be used to describe the growth kinetics of each compartment’s subpopulation, as described below.

### Tumor compartmentalization by radial distance to vasculature

We introduce a cross-sectional representation of the tumor microenvironment into compartments of shared tumor growth based on radial distance to proximal vasculature. With this radial dimension, a cylindrical model extends the cross-section to represent the growing tumor, as discussed later. The oxygen gradient maintained radially from the vasculature is parameterized and discretized to compute the numerical solution. The discretized regions define tumor cell populations with the same proliferative and dying potentials, defining *n* tumor cell compartments $T_i$, each with its own growth rate $k_{G,i}$ as a function of distance to vasculature (Fig. 1). These compartments form a coupled system of linear ordinary differential equations. It should be noted that parameter $\sigma$, or the partitioning resolution, is the compartment thickness of the radially-partitioned oxygen gradient. To model subpopulations at the highest resolution, $\sigma$ is set to approximate a single-cell diameter at 10 $\mu$m. Along with a necrotic cuff of 100 $\mu$m, this discretization yields $n=10$ distinct compartments or tumor regions. The following formulation is used to define the radial relationship between compartments and their growth rates:

#### Definition 1

The level set corresponding to compartment $T_i$ of a real-valued function *f* of *m* variables is defined as a set of the form:

$$\begin{aligned} T_i(f) = \{ (x_1,x_2,\ldots ,x_m) \; \vert \; f(x_1,x_2,\ldots ,x_m) = i \} \end{aligned}$$

where $$i \in {\mathbb {Z}}$$.

In order to parameterize the system, points in ${\mathbb {R}}^3$ are mapped to cylindrical coordinates, giving rise to the spatial dimension of radial distance to proximal vasculature, *r*. Accordingly, the net tumoral growth rate $k_G$ is defined as a function of *r*:

5 $$\begin{aligned} k_{G,(x,y,z)} = k_G(x,y,z) = k_G(r) = k_{G,r} \end{aligned}$$

where the coordinate (*x*, *y*, *z*) is a point in ${\mathbb {R}}^3$ and $r \triangleq \sqrt{x^2 + y^2}$ is a point along the now parameterized radius. By using the above parameterization, Definition (1), and the assumption that cells will share a similar growth pattern locally, the system is descretized by level sets that define distance-based oxygenation regions of the microenvironment cross-section’s constituent tumor regions and central vessel:

6 $$\begin{aligned} T_i(f) = \{ r \; \vert \; f(r) = i \} \quad \text {s.t.} \quad f = \lceil \frac{r}{\sigma } \rceil \end{aligned}$$

where $\lceil \cdot \rceil$ is the ceiling function and *f* is a discretizing function that maps cells of a radial distance to a discrete region, or concentric shell of the tumor. As discussed in more detail in the following sections, the $i{\text {th}}$ tumor cell compartment ($T_i$) is assigned a level set that defines its corresponding volume $v_i$ and net growth rate $k_{G,i}$. Together, these parameters encode for the distinct tumor regions to form a multi-compartment system of tumor growth. With this discretization, the net growth rate is defined as a discrete function rather than its continuous counterpart in Eq. (5). Thus, parameter $k_{G,i}$ is referred to as the *local net growth rate*:

7 $$\begin{aligned} k_{G,i} = k_G(s_i) \end{aligned}$$

where $s_i$ is the radial midpoint of cylindrical compartment $T_i$. The following mapping for $s_i$ is used to calculate all compartmental rate constants and shedding weights:

8 $$\begin{aligned} s_i = \left( \frac{2 \cdot f(r)-1}{2} \right) \cdot \sigma = \left( \frac{2i-1}{2} \right) \cdot \sigma \text {.} \end{aligned}$$

This mapping is used to define compartmental rates and shedding weights based on the corresponding radial midpoint.

### Compartmental volumes

The radial parameterization and discretization of space in the microenvironment into compartments also requires a spatial definition to encode heterogeneous population growth and shedding. With a radial dimension, a cylindrical representation defined around vasculature is used. The previous subsection introduced the level set formulation to assign compartmental rates to each subpopulation based on distance to proximal vasculature; this subsection introduces geometric definitions of cylindrical shells to define compartmental volumes. These two ideas together delineate constraints for both the tumor cell subpopulations and the vasculature (denoted as *Pl* in Fig. 1).

#### Definition 2

The volume *V* of a cylinder with radius *R* is:

$$\begin{aligned} V \triangleq \pi R^{2} h \text {.} \end{aligned}$$

#### Definition 3

The *core* ${\mathcal {C}}$ of a cylinder is defined as the sub-cylindrical region starting at the origin with radius $\delta$.

#### Definition 4

The $i{\text {th}}$ concentric shell (corresponding to compartment $T_i$) of a cylinder has a volume $V_{i}$ of:

$$\begin{aligned} V_{i}&= \pi R_{i}^{2}h \quad \text {s.t.} \quad R_{i} \leqslant R \text {,} \quad \forall i=1 \ldots n \quad \text {where} \quad V_{n} = V \text {.} \end{aligned}$$

#### Definition 5

A cylinder with radius *R* and core ${\mathcal {C}}$ is said to contain *n* *equidistantly concentric shells* if its radius is uniformly partitioned by $\sigma = \frac{R}{n}$. Namely, $\sigma$ is the thickness of each cylindrical shell (i.e., associated with compartments $T_i \, \forall i = 1 \ldots n$), where the $i{\text {th}}$ concentric shell has radius:

$$\begin{aligned} R_{i} = \sum _{j=1}^{i} \sigma _{j} + \delta = i \sigma + \delta \quad \text {s.t.} \quad R_{n} = R \text {.} \end{aligned}$$

Furthermore, the shell subvolumes of $$v_{i}$$$$(\forall i = 1 \ldots n)$$ s.t. $$\sum _{i=1}^{n}v_{i} = V_{n} = V$$ are computed in the following manner:

$$\begin{aligned} v_{1}&= V_{1} = \pi R_{1}^{2}h = \pi (\sigma + \delta )^{2}h \\ v_{2}&= V_{2} - V_{1} = \pi (R_{2}^{2} - R_{1}^{2})h = \pi [(2\sigma + \delta )^{2} - (\sigma + \delta )^{2}]h = \pi (3\sigma ^{2} + 2\sigma \delta )h \\&\vdots \\ v_{n}&= V_{n} - V_{n-1} = \pi (R_{n}^{2} - R_{n-1}^{2})h = \pi [(n\sigma + \delta )^{2} - ((n-1)\sigma + \delta )^{2}]h = \pi [(2n-1)\sigma ^{2} + 2\sigma \delta ] h \text {.} \end{aligned}$$

#### Proposition 1

For a cylinder with radius *R* and equidistantly concentric shells, the following recurrence relation and solution describe corresponding subvolumes $v_{i}$:

$$\begin{aligned} v_{n}&= \left[ \frac{(2n-1)\sigma + 2\delta }{(2n-3)\sigma + 2\delta } \right] \cdot v_{n-1} \\ v_{n}&= \pi [(2n-1)\sigma ^{2} + 2\sigma \delta ] h \\ \forall n&\in {\mathbb {N}}\quad \text {and basis case:} \quad v_{1} = V_{1} = \pi R_{1}^{2}h = \pi (\sigma + \delta )^2 h \text {.} \end{aligned}$$

Given that the MVD (*d*) has been measured at approximately 60 $\mu$m in some tissue types, the radius of the average vasculature in contact with the proposed microenvironment cross-section is approximately 30 $\mu$m. This latter distance is denoted as the cylindrical model’s core, described as $\delta$ in **Definition** 3. Thus the radius of the cylindrical model is $R = \delta + \epsilon$, where $\epsilon$ is the aforementioned necrotic cuff. Refer to Fig. 1 to see a summary of spatial constraints. Using **Proposition** 1, subvolumes $v_i$ of cylindrical shells associated with compartments $T_i$ are calculated:

$$\begin{aligned} v_{i} = \pi \left[ (2i-1)\sigma ^{2} + 2\sigma \delta \right] h \text {.} \end{aligned}$$

The height of the cylindrical system $h_t$, or level of tumor vascularization, is defined as a function with respect to time. The following linear vascularization function is assumed to simulate a slow-growing tumor:

$$\begin{aligned} h_t = k_V \cdot t \end{aligned}$$

with $k_V$ denoting the rate of vascularization.

### Compartmental carrying capacities

The compartmental volumes $v_i$ help determine a compartment $T_i$’s associated physical capacity, $K_{i,t}$. This can also be thought of as a numerical cell limit before cells begin outgrowth into the next compartment, i.e., being advected towards lower-pressure necrotic regions away from the vascular source by the oncotic pressure exerted by better oxygenated, proliferative cells. This relationship can be calculated by the following expression, using parameters measured from solid tumor cellular densities:

9 $$\begin{aligned} K_{i,t} = v_i \cdot \rho \cdot p \end{aligned}$$

where $\rho$ is the approximate tumor cell density (cells/mm$^{3}$) of a solid tumor, and *p* is the percent of total tumor volume occupied by tumor cells^8^ (Table 1). Differing compartmental volumes account for differing carrying capacities. As briefly mentioned earlier with the introduction of a vascularization function $h_t$, the volume and subsequent carrying capacity of a compartment changes over time. In effect, $h_t$ extends compartmental carrying capacities as blood vessels are recruited to increase tumor vascularization.

### Compartment dynamics during tumor growth

The previous subsections fully defined tumor cell compartments $T_i$ with their own (1) effective rates of growth and shedding, (2) volumes, and (3) carrying capacities to define constraints for their subpopulations. This subsection introduces cell motility and aggregate tumor outgrowth into more distant regions. For simplicity, cell metastatic migration is neglected in this process, and cells dying at each time step do not occupy physical space toward the capacity of the compartment. Additionally, some amount of cellular movement is allowed between the compartments to account for multi-directional cell proliferation and death. With a lack of data available for cell motility, the following probabilities (and resulting fractions) of compartment or subpopulation movement are used:

$$\begin{aligned}&{\mathbb {P}}(\text {inward movement}) := 0.10 \\&{\mathbb {P}}(\text {outward movement}) := 0.05 \\&{\mathbb {P}}(\text {outward movement | 10-fold difference}) := 0.10 \end{aligned}$$

where inward movement is slightly favored due to higher oxygenation. These probabilities were manually tuned via the tumor growth model performance.

This multi-compartment system accounts for the self-interacting dynamics between subpopulations. Due to the addition of radial distance as a dimension, one must account for cellular growth that expands outward into a neighboring compartment, or oxygenation region, of the tumor. In the model, as cell populations reach their compartment’s carrying capacities, they proliferate into more necrotic compartments under the assumption that the oncotic pressure decreases towards necrotic regions. Accordingly, the index notation $i=1 \ldots n$ is employed to signify the $i{\text {th}}$ tumor compartment $T_i$ and its corresponding proliferating population $P_{i,t}$. Outgrowth into the $i+1{\text {th}}$ compartment from the neighboring $i{\text {th}}$ compartment depends on the local spatial capacity of the $i{\text {th}}$ compartment, which is denoted as $K_{i,t}$. More precisely, the $i{\text {th}}$ compartment’s proliferating population must exceed that of its own capacity such that $P_{i,t} > K_{i,t}$. If this condition holds true, excess cells created in the current time-step *t* are henceforth contributing to the proliferating population of the $i+1{\text {th}}$ compartment, $P_{i+1,t}$ (Table 2). This outgrowth (i.e., compartment overflow) at day *t*, $O_{i+1,t}$, is defined as a recursive difference equation:

10 $$\begin{aligned} O_{i+1,t} {:}=\mathbb {1}_{>1} (i+1) \cdot \mathbb {1}_{>K_{i,t}} (P_{i,t} ) \cdot [P_{i,t}-K_{i,t}] \end{aligned}$$

This outgrowth term is added to $P_{i,t+1}$, compartment $T_i$’s proliferative population at time $t+1$ to yield a recursive definition of proliferative tumor population per compartment $T_i$. With this spatiotemporal dynamism, each compartment is also governed by its corresponding local net growth rate $k_{G,i}$:

11 $$\begin{aligned} P_{i,t+1} \approx \left[ (1+k_{G,i} \Delta t) \cdot P_{i,t} \right] + O_{i,t+1} \end{aligned}$$

**Table 2:** Model variables.

Variable	Description
$P_{i}(t)$	Proliferative population of compartment $T_{i}$at continuous time *t* (cell)
$P_{i,t}$	Proliferative population of compartment $T_{i}$at discrete time *t* (cell)
$D_{i,t}$	Dead cell population of compartment $T_{i}$ at discrete time *t* (cell)
$O_{i,t}$	Cell overflow from compartment $T_{i}$ to $T_{i+1}$ (cell)
$h_{t}$	Height of compartments (and cylindrical model) at discrete time *t*
$K_{i,t}$	Carrying capacity of compartment $T_{i}$at discrete time *t* (cells)
$u_{i}(t)$	Protein outflux from compartment $T_{i}$ at continuous time *t* (U/day)
$u_{i,t}$	Protein outflux from compartment $T_{i}$ at discrete time *t* (U/day)
*q*(*t*)	Protein mass in plasma at continuous time *t* (U)
$q_{\mathrm {t}}$	Protein mass in plasma at discrete time *t* (U)

### Heterogeneous intratumoral protein shedding

Consistent with previously established approaches^8^, protein shedding is represented as a relationship directly proportional to the number of cells in the system. Accordingly, the $i{\text {th}}$ compartmental outflux at time *t*, $u_i(t)$, is defined as a distance-based relationship between the number of tumor cells and the corresponding compartment’s population and shedding values:

12 $$\begin{aligned} {\left\{ \begin{array}{ll} u_{i,t} \approx w_i \Psi \cdot \frac{D_{i,t}}{\Delta t} &{} \text {if} \, EC=0 \\ u_i(t) = w_i \Phi \cdot P_i(t) &{} \text {if} \, EC=1 \end{array}\right. } \end{aligned}$$

where $D_{i,t} / \Delta t$ is the dead tumor cell population for tumor compartment $T_i$ per day *t*, *EC* is the cellular localization of the protein ($EC=1$ denotes the extracellular domain and $EC=0$ denotes the non-extracellular domain), $\Psi$ is the protein-specific contribution per cell during necrosis, $\Phi$ is the protein-specific shedding rate per cell per day, and $w_i$ is the distance-based weight for the $i{\text {th}}$ tumor compartment, $T_i$ (Table 1). Note that $u_{i,t}$ is the $i{\text {th}}$ compartment’s protein influx into the plasma at discrete day *t*. For the sake of simplicity, $w_i$ is approximated as a 1-dimensional diffusivity relationship with distance from vasculature:

13 $$\begin{aligned} w_i = \frac{1}{\sqrt{s_i}} \end{aligned}$$

Finally, extracellular protein shedding is accounted for by active shedding from proliferating cells, while non-extracellular protein shedding is directly proportional to the instantaneous necrotic population at time *t*.

### Heterogeneous plasma protein influx

Extracellular and non-extracellular protein mass in plasma, *q*(*t*) or $q_t$, is modeled with the following ordinary difference and differential equations:

14 $$\begin{aligned}&\frac{\hbox {d}q(t)}{\hbox {d}t} = \sum _{i=1}^n u_i(t) + u_H - k_E \cdot q(t) \end{aligned}$$

15 $$\begin{aligned}&\implies q_{t+1} \approx \sum _{i=1}^n u_{i,t} \Delta t + u_H \Delta t + (1 - k_E \Delta t) \cdot q_t \end{aligned}$$

where $k_E$ is the elimination rate of protein from the plasma, which encompasses protein degradation, excretion, clearance, etc. Equation (14) is approximated numerically as a difference equation in Eq. (15) using the Euler method. Parameter $q_0$ is the basal plasma protein mass, and relatedly, $u_H$ is the daily rate constant of influx of protein mass from healthy cells. It should be noted that when multiplied by $\Delta t$, the rate $u_H$ becomes an influx value at each time step. Furthermore, in experimental settings with tumor xenografts in mouse models, $u_H$ is assumed to be zero-valued since the generated human proteins of interest are non-endogenous.

### Estimation of model parameter values

In order to build mappings between distance to vasculature *r* and net growth rate $k_{G,i}$, data from both oxygen diffusion in tissue and *in vitro* tumor cell birth and death rates under varying oxygen conditions are used^20^. A compartment $T_i$’s tumor growth parameters $k_{G,i}$, $k_{B,i}$, and $k_{D,i}$ is assumed linear with respect to oxygen concentration and estimated with the following equations:

16 $$\begin{aligned} C(r)&\triangleq C_0 \cdot 0.5^{r/r_{1/2}} \end{aligned}$$

17 $$\begin{aligned} {\widehat{k}}_{\{\cdot \}, r}&:= m_{\{\cdot \}} \cdot C(r) + b_{\{\cdot \}} \end{aligned}$$

where $C_0 \triangleq C(0)$ is the volumetric concentration of oxygen at vasculature, $r_{1/2}$ is the distance half-life of oxygen, *m* is the slope describing the change in rates over the change in oxygenation, and *b* is the minimum measured rate value. Equation (16) assumes an exponential decay relationship^20^ mapping radial distance from a proximal vasculature point *r*, to the volumetric concentration of oxygen *C*, thereby creating a concentration gradient over the domain $r \in [0,\epsilon ]$. Equation (17) assumes a linear relationship that maps the volumetric concentration of oxygen *C* to the net growth, birth, or death rate. Specifically, a linear interpolation is employed on birth and death rate data collected from cell cultures exposed to $1\%$ and $20\%$ oxygen to estimate *n* sets of birth, death, and net growth rates for the compartments midpoints $s_i$ as seen in Eq. (8) with the level set formulation. The resulting rates can be found in Table 1. A visualization of the birth, death, and net growth rates can be found in Fig. 2.

### Software implementation

The model was simulated using Python. Due to the coupled nature of tumor growth and protein shedding, these two phenomena were simulated in series, each with its own sets of parameters. The parameter set corresponding to tumor growth relies on the cell or tissue type, while the parameter set corresponding to protein shedding relies on the proteins of interest.

## Data Availability

The source code for the model is available at https://github.com/gmachiraju/MulticompartmentTumors.
